# A dual tracer [^11^C]PBR28 and [^18^F]FDG microPET evaluation of neuroinflammation and brain energy metabolism in murine endotoxemia

**DOI:** 10.1186/s42234-022-00101-2

**Published:** 2022-11-30

**Authors:** Santhoshi P. Palandira, Joseph Carrion, Lauren Turecki, Aidan Falvey, Qiong Zeng, Hui Liu, Tea Tsaava, Dov Herschberg, Michael Brines, Sangeeta S. Chavan, Eric H. Chang, An Vo, Yilong Ma, Christine N. Metz, Yousef Al-Abed, Kevin J. Tracey, Valentin A. Pavlov

**Affiliations:** 1 The Feinstein Institutes for Medical Research, Northwell Health, Manhasset, NY USA; 2 Donald and Barbara Zucker School of Medicine at Hofstra/Northwell, Hempstead, NY USA

**Keywords:** Murine endotoxemia, Brain, Neuroinflammation, Micropet imaging, Microglia, Brain metabolism, Conjunction analysis, [^11^C]PBR28, [^18^F]FDG

## Abstract

**Background:**

Brain metabolic alterations and neuroinflammation have been reported in several peripheral inflammatory conditions and present significant potential for targeting with new diagnostic approaches and treatments. However, non-invasive evaluation of these alterations remains a challenge.

**Methods:**

Here, we studied the utility of a micro positron emission tomography (microPET) dual tracer ([^11^C]PBR28 – for microglial activation and [^18^F]FDG for energy metabolism) approach to assess brain dysfunction, including neuroinflammation in murine endotoxemia. MicroPET imaging data were subjected to advanced conjunction and individual analyses, followed by post-hoc analysis.

**Results:**

There were significant increases in [^11^C]PBR28 and [^18^F]FDG uptake in the hippocampus of C57BL/6 J mice 6 h following LPS (2 mg/kg) intraperitoneal (i.p.) administration compared with saline administration. These results confirmed previous postmortem observations. In addition, patterns of significant simultaneous activation were demonstrated in the hippocampus, the thalamus, and the hypothalamus in parallel with other tracer-specific and region-specific alterations. These changes were observed in the presence of robust systemic inflammatory responses manifested by significantly increased serum cytokine levels.

**Conclusions:**

Together, these findings demonstrate the applicability of [^11^C]PBR28 - [^18^F]FDG dual tracer microPET imaging for assessing neuroinflammation and brain metabolic alterations in conditions “classically” characterized by peripheral inflammatory and metabolic pathogenesis.

**Supplementary Information:**

The online version contains supplementary material available at 10.1186/s42234-022-00101-2.

## Background

Brain neuronal dysfunction and neuroinflammation (inflammation in the central nervous system, CNS) are traditionally attributed to Alzheimer’s disease and other neurodegenerative diseases (Heneka et al, 2015; Metz & Pavlov, 2021; Streit et al, 2004; Leng & Edison, 2021). However, brain dysfunction, including neuroinflammation has been also documented in conditions “classically” characterized by peripheral immune and metabolic dysregulation, including endotoxemia and sepsis (Pavlov & Tracey, 2015). Endotoxemia, induced by peripheral lipopolysaccharide (LPS) administration provides a standardized model to study innate immune responses and the cardiometabolic effects of systemic cytokine release and inflammation both in animals and humans (Patel et al, 2015; Buras et al, 2005). Endotoxemia has been also used to study immune and cardiometabolic dysregulation with some relevance to sepsis - a devastating condition defined as life-threatening organ dysfunction caused by a dysregulated host response to infection (Singer et al, 2016). The brain is profoundly affected in endotoxemia, and sepsis, and *sepsis associated encephalopathy* - a severe brain dysfunction caused by systemic inflammation in the absence of direct brain infection, has been documented (Gofton & Young, 2012; Meneses et al, 2019). Sepsis associated encephalopathy is linked to significantly higher mortality and early diagnosis is key for improving the outcome (Gofton & Young, 2012; Sprung et al, 1990). Among other factors in the brain, neuroinflammation and metabolic derangements contribute to the development of sepsis associated encephalopathy (Gofton & Young, 2012; Meneses et al, 2019). Therefore, real time evaluation of brain function using non-invasive approaches in these conditions is important for designing new diagnostic approaches and evaluation of current and potential treatments within the scope of Bioelectronic Medicine (Pavlov & Tracey, 2022).

Positron emission tomography (PET) provides a non-invasive approach for imaging the brain and studying functional alterations including neuroinflammation and altered energy metabolism in disease settings. Microglia are a major cell type with immune function in the CNS/brain and a key driver of neuroinflammation (Streit et al, 2004). Astrocytes, the most abundant cell type in the CNS/brain also have key roles in neuroinflammation (Giovannoni & Quintana, 2020). Microglia and astrocytes provide structural, trophic, and metabolic support to neurons and are active modulators of neuronal synaptic interaction and neuronal plasticity; changes in microglia and astrocyte functional states can result in neuronal synaptic reorganization, impaired neuronal survival and altered brain function (Garden & Möller, 2006; Hanisch & Kettenmann, 2007; Yirmiya & Goshen, 2011; Chen & Swanson, 2003). The use of specific radiotracers targeting translocator protein (TSPO), formerly known as peripheral benzodiazepine receptor (PBR) has been broadly utilized in PET evaluation of neuroinflammation (Pannell et al, 2020). PBR is an 18-kDa mitochondrial outer membrane translocator protein, which is widely expressed by many cell types, but in the CNS, it is exclusively localized in microglia, astrocytes, and macrophages (Pannell et al, 2020; Lavisse et al, 2012; Chen & Guilarte, 2008). While under normal physiological conditions TSPO (PBR) expression in the CNS is minimal, it is highly upregulated during neuroinflammation associated with brain injury and many neurodegenerative conditions (Pannell et al, 2020; Lavisse et al, 2012; Chen & Guilarte, 2008). The TSPO ligand [^11^C]-peripheral benzodiazepine receptor, [methyl-^11^C]N-acetyl-N-(2-methoxybenzyl)-2-phenoxy-5-pyridinamine ([^11^C]PBR28), has been successfully used as a PET radiotracer for assessing neuroinflammation in rodents (Walker et al, 2015; Mirzaei et al, 2016), non-human primates (Brown et al, 2007; Hannestad et al, 2012), and humans (Kreisl et al, 2009; Alshikho et al, 2018; Sandiego et al, 2015; Toppala et al, 2021; Gershen et al, 2015). [^18^F] Fluoro-2-deoxy-2-D-glucose [^18^F]FDG is a glucose analog that is taken up by living cells via cell membrane glucose transporters and subsequently phosphorylated with hexokinase inside most cells; it is not metabolized and does not exit cells. [^18^F]FDG is a widely used radiotracer for the determination of localized metabolic alterations in the body and has been utilized in the diagnosis and therapy monitoring in various disorders, including cancer, and infectious and inflammatory diseases (Chacko et al, 2017; Glaudemans et al, 2013; Fletcher et al, 2008). PET [^18^F]FDG imaging is also frequently used to assess brain metabolic activity (Sibson et al, 1998; Rocher et al, 2003; Vo et al, 2014; Uluğ et al, 2011; Vo et al, 2015). In the brain, in addition to neurons, experimental evidence indicates that [^18^F]FDG PET signal can be driven by astrocytes (Zimmer et al, 2017). It is also documented that microglia increase their metabolic output during inflammation (Baik et al, 2019; Lauro & Limatola, 2020). Therefore, PET [^18^F]FDG imaging provides information about energy metabolism based on glucose utilization by neurons, astrocytes, and microglia.

We and others have previously shown brain microglial activation, neuroinflammation and metabolic and neurotransmitter system changes in murine models of endotoxemia or cecal ligation and puncture-induced sepsis using invasive and terminal approaches (Silverman et al, 2015; Zaghloul et al, 2017; Hoogland et al, 2015; Qin et al, 2007; Buttini et al, 1996). Here, we investigated the application of dual tracer [^11^C]PBR28 and [^18^F]FDG microPET imaging in endotoxemic mice by utilizing an advanced conjunction analysis of the imaging data generated. This methodology involving conjunction analysis provides the advantage of characterizing common brain regions with overlapping neuroinflammatory and metabolic alterations in addition to detecting changes in the brain uptake of each tracer separately. We observed the presence of overlapping increases in radioligand uptake in the hippocampus, the thalamus, and the hypothalamus, and radiotracer-specific regional alterations, in parallel with increased serum cytokine levels. These observations demonstrate the utility of this dual tracer microPET approach with conjunction analysis to non-invasively examine metabolic activation and neuroinflammation, which have been previously reported in postmortem brain analyses. This methodology can be applied in future studies evaluating brain alterations in preclinical and clinical scenarios.

## Methods

### Animals

Experiments were performed in accordance with the National Institutes of Health guidelines, and all experimental procedures with animals were approved by the Institutional Animal Care and Use Committee and the Institutional Biosafety Committee of the Feinstein Institutes for Medical Research before humane experimentation. C57BL/6 J male mice (13–15 weeks old, Jackson Laboratory) were maintained at room temperature on a 12-h light/dark cycle with free access to food and water.

### Endotoxemia

Mice were injected with lipopolysaccharide (LPS, endotoxin) (2 mg/kg, i.p.) (*n* = 6) or sterile saline (i.p.) alone as the vehicle control (*n* = 6). Six hours after LPS or saline injection mice underwent microPET imaging as described below. In parallel experiments, mice were administered with saline or LPS i.p. and euthanized at the same time point – 6 h, and blood was collected via cardiac puncture and processed for serum cytokine analyses.

### MicroPET imaging and analyses

MicroPET imaging was performed using the Inveon® MicroPET imaging system (Siemens) at 6 h post endotoxin or vehicle administration. Briefly, upon arrival to the imaging suite, animals were acclimated for one hour and then anesthetized with 2–2.5% isoflurane mixed in oxygen and the tail vein was cannulated using a 30 G custom catheter. [^18^F]FDG and [^11^C]PBR28 are routinely synthesized onsite at the PET imaging facility at the Feinstein Institutes and delivered directly into the microPET suite. Approximately 0.5 mCi of [^11^C]PBR28 (in 0.2 ml) was slowly injected via the tail vein with the simultaneous start of a 60-min dynamic imaging acquisition, followed by a 10-min transmission scan on a Siemens Inveon MicroPET. 1.5 h after the [^11^C]PBR28 scan, ~ 0.5 mCi of [^18^F]FDG (in 0.3 ml) was injected i.p. with 35–40 min allowed for uptake of the tracer followed by a 10 min static scan. Brain images were acquired and reconstructed using Inveon Acquisition workflow (IAW 1.5) and three-dimensional ordered subsets-expectation maximization (3D-OSEM) reconstruction with attenuation correction. After reconstruction, raw images were bounding box aligned, skull stripped, and dose and weight corrected. [^18^F]FDG scans from each animal were registered to an [^18^F]FDG template (Schiffer et al, 2007) and then to a common MRI template (Ma et al, 2005), both of which were aligned within Paxinos and Franklin anatomical space, using Pixel-Wise Modeling (PMOD) 4.0 Software. The rigid transformations from the template-aligned [^18^F]FDG scans were then applied to the corresponding [^11^C]PBR28 scans for each animal using statistical parametric mapping (SPM)5 mouse toolbox within MATLAB. Regarding the [^11^C]PBR28 scan, the final 10 frames of each scan (final 22 mins of dynamic scan) were averaged and used for analysis. Images were smoothed with an isotropic Gaussian kernel FWHM (full width at half maximum) 0.56 mm at all directions.

To identify regions in anatomical space with significant differences between LPS administered and saline administered mice in both [^18^F]FDG and [^11^C]PBR28 tracers, we performed whole brain voxel-wise searches with conjunction analysis using SPM-Mouse software (The Wellcome Centre for Human Neuroimaging, UCL Queen Square Institute of Neurology, London, UK, https://www.fil.ion.ucl.ac.uk/spm/ext/#SPMMouse) (Sawiak et al, 2009). The conjunction analysis identified the group effects common to the dual tracer, in which the contrasts, testing for a group effect, are specified separately for each tracer. These contrasts are thresholded at a common threshold and combined to give the conjunction. This combination is on a voxel-by-voxel basis, and a new contrast that tests for the conjunction is created. This model was setup with full factorial analysis (2 × 2) with 2 tracers ([^18^F]FDG and [^11^C]PBR28) and 2 groups (LPS and saline). Inter-subject variability in imaging data was accounted by dividing each PET scan by its global mean value in comparison with the use of cerebellum as a normalization factor. Group differences were considered significant at a voxel-level threshold of *P* < 0.005 for conjunction analysis and *P* < 0.001 for individual analysis with a cluster cutoff of 500 voxels. We identified the significant conjunction clusters, in which both [^18^F]FDG and [^11^C]PBR28 values increase or decrease in LPS-administered mice relative to saline-administered mice. We also checked if there are significant clusters, in which values are increased in one tracer and decreased in the other in LPS relative to saline mice or vice versa. We also performed whole brain voxel-wise searches separately for each tracer to validate the results from the conjunction analysis. Individual data from each significant clusters (in Paxinos and Franklin anatomical space) (Paxinos & Franklin, 2019) were identified throughout the whole-brain searches and were measured with post-hoc volume-of-interest (VOI) analyses using in-house MAPLAB scripts. [^18^F]FDG and [^11^C]PBR28 values for the LPS- and saline-treated mice were visualized to evaluate overlapping data and potential outlier effects.

### Statistical analysis

All data are presented as the mean ± SEM. Data analysis was performed using GraphPad Prism software 9.0. Cytokine analysis was performed using the unpaired Mann-Whitney test and *P* < 0.05 was considered significant. In post-hoc analysis of microPET data, values for each significant cluster were similarly compared between the two groups using the unpaired Mann-Whitney test and *P* < 0.05 was considered significant.

## Results and discussion

**Fig. 1:**
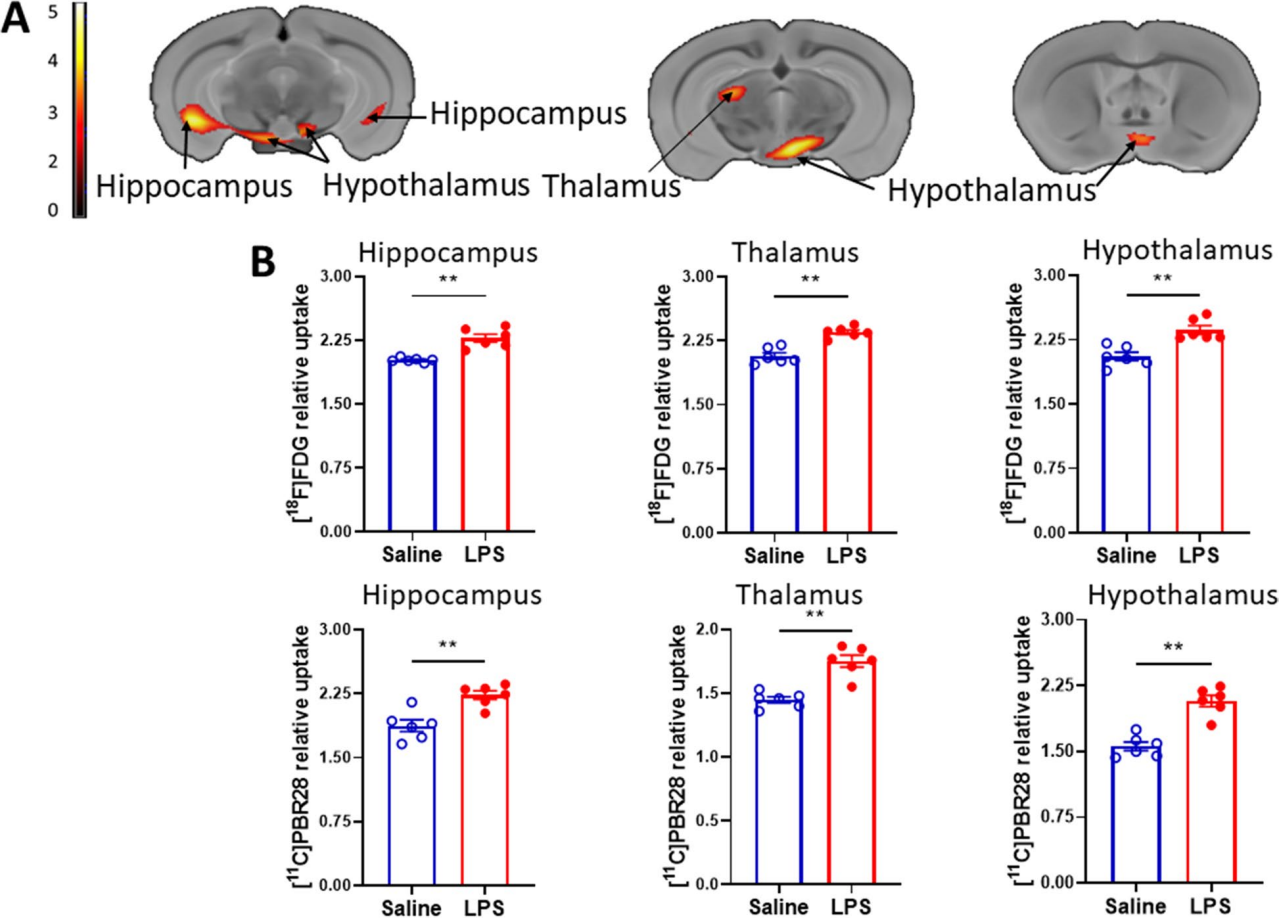
Brain regions with overlapping increases in [^11^C]PBR28 and [^18^F]FDG uptakes during endotoxemia. **A** Dual tracer conjunction increases in metabolism and microglial activation (TSPO binding). Brain coronal sections of statistically significant clusters (color bar represents t-value height, extent threshold T = 2.85), overlaid onto MRI atlas for visualization. Statistically significant clusters (*P* < 0.005) show overlapping regions of increased uptake of [^11^C]PBR28 and [^18^F]FDG in the hippocampus, the thalamus, and the hypothalamus in LPS administered (*n* = 6) vs saline administered (*n* = 6) mice (see Table 1 for stereotaxic coordinates). **B** Post-hoc analysis for the same statistically significant increases in metabolic activity [^18^F]FDG and microglial activation [^11^C]PBR28 (TSPO binding) in saline (*n* = 6) and LPS (*n* = 6) mice. **P = 0.002, **P = 0.004 (hippocampus - [^11^C]PBR28). See Methods for details

**Fig. 2:**
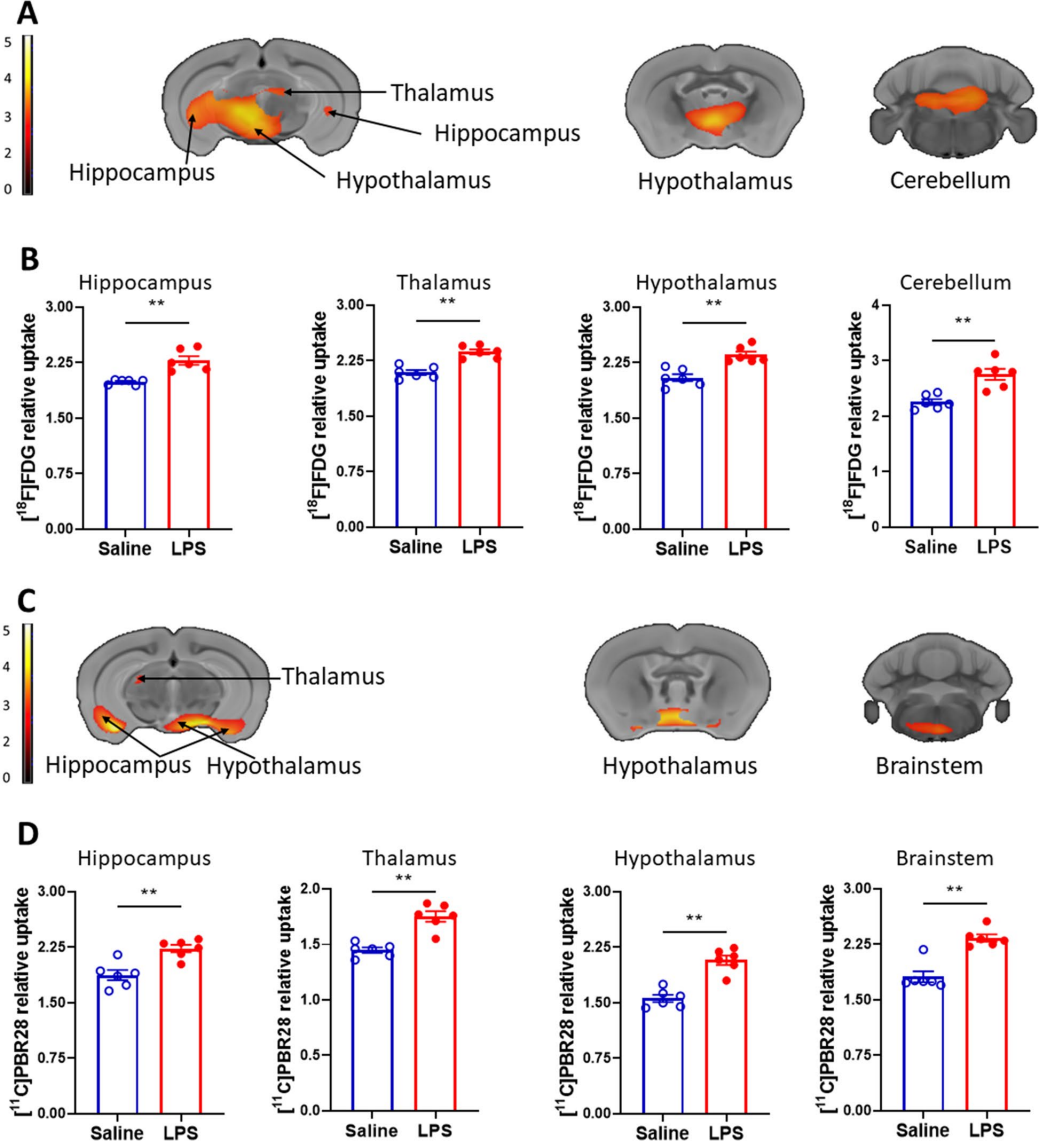
Brain regions with individual increases in [^11^C]PBR28 and [^18^F]FDG uptake during endotoxemia. **A** Increased [^18^F]FDG uptake in the hippocampus, the thalamus, the hypothalamus, and the cerebellum shown in coronal sections in LPS administered (*n* = 6) vs saline administered (*n* = 6) mice (see Table 1 for specific stereotaxic coordinates). SPM clusters (color bar represents t-value height, cutoff threshold T = 3.55) overlaid onto MRI atlas for visualization. Statistically significant clusters (*P* < 0.001). **B** Post-hoc analysis of [^18^F]FDG tracer uptake in the same groups (saline, *n* = 6 and LPS, *n* = 6) of mice. **P = 0.002. **C** Increased [^11^C]PBR28 metabolic activity in the hippocampus, the thalamus, the hypothalamus, and the brainstem shown in coronal sections in LPS administered (*n* = 6) vs saline administered (*n* = 6) mice (see Table 1 for stereotaxic coordinates). SPM clusters (color bar represents t-value height, cutoff threshold T = 3.55). Statistically significant clusters (*P* < 0.001) overlaid onto MRI atlas for visualization. **D** Post-hoc analysis of [^11^C]PBR28 uptake in the same groups (saline, *n* = 6 and LPS, *n* = 6) of mice. **P = 0.002, **P = 0.004 (hippocampus). See Methods for details

**Fig. 3:**
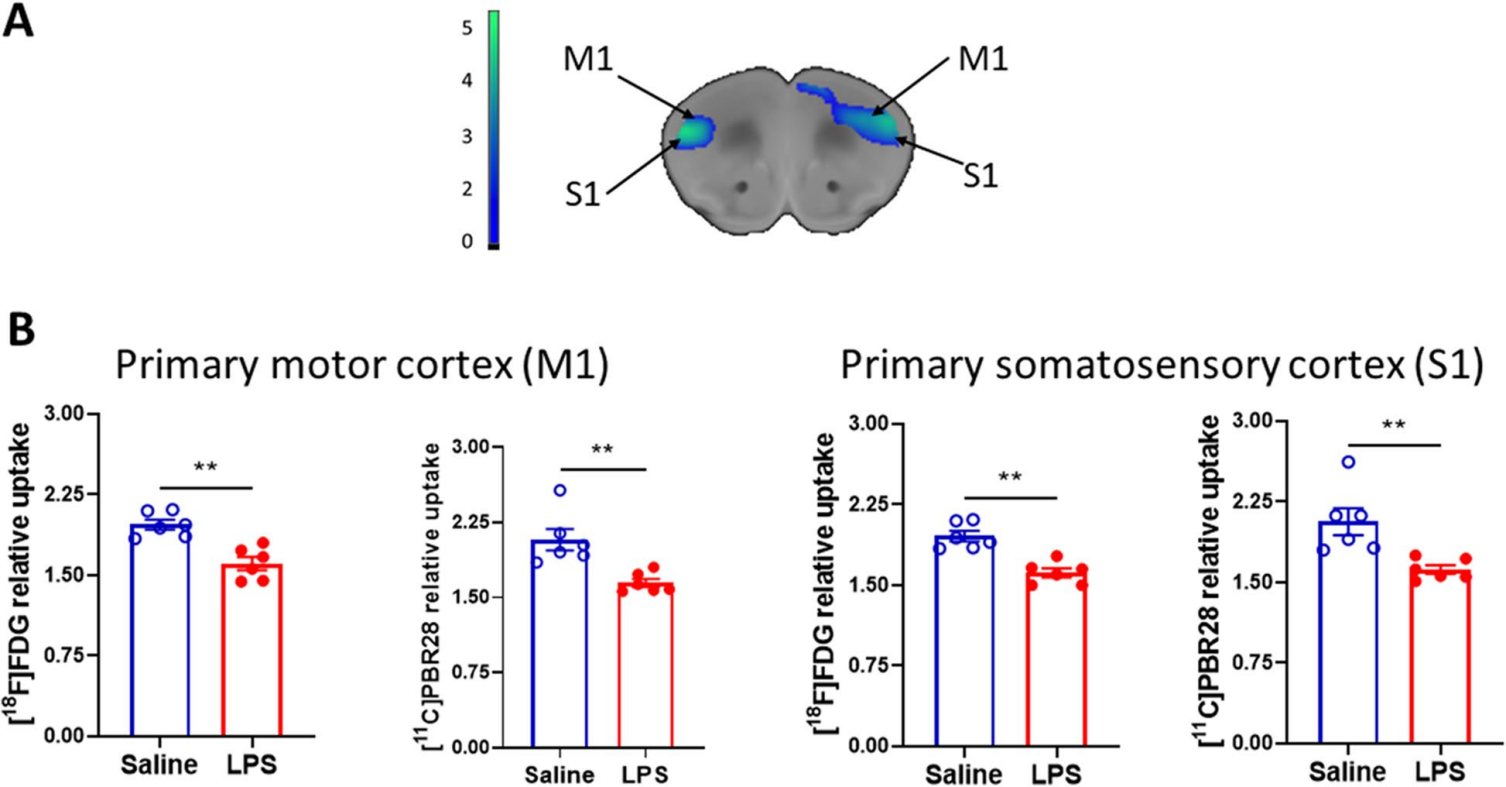
Brain regions with overlapping significantly decreased [^11^C]PBR28 and [^18^F]FDG uptake during endotoxemia. **A** Simultaneous (dual tracer) uptake decreases in primary and somatosensory motor cortices in LPS administered (*n* = 6) vs saline administered (*n* = 6) mice. Dual tracer decreases in metabolism and TSPO binding shown with SPM cluster overlaid onto MRI atlas for visualization (color bar represents t-value height, cutoff threshold T = 2.85). **B** Individual [^18^F]FDG and [^11^C]PBR28 decreases in primary and somatosensory motor cortices were subjected to post-hoc analysis in the same groups (saline, *n* = 6 and LPS, *n* = 6) of mice. **P = 0.002. See Methods for details

Brain dysfunction including immune and metabolic changes have been described in animals with endotoxemia and CLP sepsis (Silverman et al, 2015; Zaghloul et al, 2017; Hoogland et al, 2015; Qin et al, 2007). We and others have previously shown that peripheral (i.p.) administration of LPS in mice results in neuroinflammatory alterations, including characteristic morphological changes in microglia, indicative of activation in the hippocampus and other brain areas (Silverman et al, 2015; Hoogland et al, 2015; Qin et al, 2007). Here we examined whether neuroinflammation in these brain areas during endotoxemia can be also detected in live mice using a dual tracer microPET imaging subjected to an advanced conjunction analysis. We injected groups of male C57Bl/6 mice with LPS (2 mg/kg) or vehicle (saline) (i.p.) and confirmed the presence of systemic inflammation manifested by significantly increased serum cytokine (TNF, IL-6, IL-1β, and IL-10) levels at 6 h (Supplementary Fig. 1). Other cohorts of male C57Bl/6 mice were injected with vehicle or the same dose of LPS 6 h prior to acquiring microPET imaging utilizing [^11^C]PBR28 (~ 0.5 mCi), followed by [^18^F]FDG (0.5 mCi) as described in detail in Material and Methods. Applying an advanced conjunction comparative analysis (endotoxemic vs control mice) revealed a significantly increased simultaneous uptake of [^11^C]PBR28 and [18F]FDG in the hippocampus (Fig. 1A, B) in line with previous immunohistochemical observations showing microglial activation in this brain area (Silverman et al, 2015; Qin et al, 2007). In addition, simultaneous dual tracer uptake increases were observed in the hypothalamus and the thalamus (Fig. 1A, B). The individual analyses of [^11^C]PBR28 and [^18^F]FDG uptake (endotoxemic vs saline administered control mice) confirmed increases in each tracer’s uptake in the hippocampus, the hypothalamus, and the thalamus (Fig. 2A, B, C, D). In addition, region-specific increases were also observed; [^18^F]FDG uptake was significantly increased in the cerebellum (Fig. 2A, B), while [^11^C]PBR28 uptake was significantly increased in the brainstem (Fig. 2C, D). The SPM conjunction and individual analyses revealed specific patterns of increased brain [^18^F]FDG and [^11^C]PBR28 uptake as shown in Table 1. Please include Table 1 here. Applying conjunction analysis also revealed specific decreases in [^18^F]FDG and [^11^C]PBR28 uptake in the primary motor and somatosensory cortices (endotoxemic vs control mice) as shown in Fig. 3A, B. These decreases were further confirmed by individual analysis of [^18^F]FDG and [^11^C]PBR28 uptake as shown in Supplementary Fig. 2. All results generated using the global mean of normalization were also confirmed using cerebellum value as a reference for normalization (data not shown). These results indicate that peripheral administration of LPS, which causes systemic inflammation manifested by significant increases in circulating cytokine levels, also results in neuroinflammation and brain metabolic alterations, which can be determined using microPET dual tracer imaging.

**Table 1:** Specific brain regions with significant [^18^F]FDG and [^11^C]PBR28 uptake increases*

Radiotracer/Brain region	Brain coordinates**			Relative radiotracer uptake (controls) (mean ± SD)	Relative radiotracer uptake (LPS) (mean ± SD)
	X (mm)	Y (mm)	Z (mm)		
Dual [^18^F]FDG and [^11^C]PBR28 uptake					
Paraventricular hypothalamus anterior parvicellular	0.2	−0.5	−4.5	[^18^F]FDG = 2.06 ± 0.11 [^11^C]PBR28 = 1.56 ± 0.11	[^18^F]FDG = 2.36 ± 0.11 [^11^C]PBR28 = 2.07 ± 0.14
CA1 Hippocampus pyramidal cell layer	−3.3	− 3.5	− 4.1	[^18^F]FDG = 2.02 ± 0.03 [^11^C]PBR28 = 1.87 ± 0.15	[^18^F]FDG = 2.28 ± 0.10 [^11^C]PBR28 = 2.23 ± 0.11
CA1 Hippocampus pyramidal cell layer	3.4	−3.3	−4.0		
LP Thalamic nucleus laterocaudal	−1.9	− 2.8	− 2.6	[^18^F]FDG = 2.07 ± 0.08 [^11^C]PBR28 = 1.45 ± 0.05	[^18^F]FDG = 2.34 ± 0.06 [^11^C]PBR28 = 1.75 ± 0.11
[^18^F]FDG uptake					
Glomerular layer olfactory	0.0 0.0 −0.2	4.4 5.2 5.5	−2.9 − 3.4 − 2.8	2.14 ± 0.10	2.71 ± 0.26
Periaqueductal grey	−2.3	− 2.8	−2.9	2.12 ± 0.07	2.50 ± 0.21
CA1 Pyramidal layer hippocampus	3.3	−3.0	−3.4	1.99 ± 0.03	2.28 ± 0.13
Red nucleus parvicell part	− 0.6	−3.2	− 3.8	2.29 ± 0.21	2.83 ± 0.14
Posterior thalamic nucleus	−1.0	−3.4	− 3.4	2.09 ± 0.08	2.37 ± 0.07
[^11^C]PBR28 uptake					
Tuberal region lateral hypothalamus	0.9	−0.9	−5.2	1.56 ± 0.11	2.07 ± 0.14
Medial amygdalar nucleus posterodorsal	1.8	−1.4	−5.2	1.85 ± 0.10	2.34 ± 0.10
Parasubthalamic nucleus	1.3	−2.1	−5.0	1.45 ± 0.05	1.75 ± 0.11
Caudomedial entorhinal	3.5 3.2	−5.1 − 5.1	−4.1 − 4.6	1.87 ± 0.15	2.23 ± 0.11

*Increased tracer uptake was identified using statistical parametric mapping (SPM5) with *P* < 0.005 in the conjunction analysis and *P* < 0.001 in the individual tracer uptake analysis (with an extent threshold of t = 500 voxels), comparing saline administered mice (*n* = 6) vs LPS administered mice (*n* = 6)

**According to (Paxinos & Franklin, 2019), X (Mediolateral), Y (Anteroposterior), Z (Dorsoventral)

[^18^F]FDG and [^11^C]PBR28 have been previously used together in microPET imaging studies of aortic aneurysm inflammation in rats (English et al, 2014) and β-amyloidosis associated inflammation in lean and obese mice (Barron et al, 2016). However, to the best of our knowledge this study is one of the first to utilize a [^18^F]FDG and [^11^C]PBR28 dual tracer imaging combined with a conjunction analysis to assess the impact of peripheral systemic inflammation on the brain.

Microglia are a major cell type with immune function in the CNS/brain and microglial activation drives neuroinflammatory processes (Streit et al, 2004; Borst et al, 2019). Astrocytes are also involved in neuroinflammation (Giovannoni & Quintana, 2020; Chen & Swanson, 2003; Bellaver et al, 2018). There is also a link between microglial activation and induction of a subtype of reactive astrocytes that is mediated through microglial secretion of TNF and other pro-inflammatory molecules (Liddelow et al, 2017). This leads to a disruption of the homeostatic relationship between astrocytes and neurons in which astrocytes promote neuronal survival and synaptogenesis, and these reactive astrocytes (designated as A1) induce neuronal death (Liddelow et al, 2017).

Brain [^18^F]FDG uptake determined using PET imaging has been traditionally viewed as a proxy of neuronal activity and its alterations in disease states, including neurodegenerative diseases (Mosconi et al, 2008). However, there is experimental evidence that glucose utilization by astrocytes also determines part of the [^18^F]FDG PET signal and [^18^F]FDG PET signal also reflects astrocyte activity (Zimmer et al, 2017). Enhanced [^18^F]FDG PET signal has been also corelated with microglial activation, for instance in mouse models of Alzheimer’s disease (Brendel et al, 2016; Poisnel et al, 2012) and in experimental neuroinflammatory conditions such as murine amyloidosis (Xiang et al, 2021). Of note, pharmacological depletion of microglia abrogates the increase in [^18^F]FDG uptake in mice with amyloidosis (Xiang et al, 2021). The clinical translatability of these findings was strengthened by observations showing good correlation between microglial activity and [^18^F]FDG uptake determined using PET (Xiang et al, 2021). In view of these previous findings, our results obtained by applying conjunction analysis and individual tracer verification indicate that the increases in [^18^F]FDG and [11C]PBR28 uptake in the hippocampus, thalamus and hypothalamus reflect brain energy metabolism alterations with neuronal, microglial and astrocytic contributions. This is supported by the individual increases in [^18^F]FDG and [11C]PBR28 uptake in these brain areas. However, our observation that [11C]PBR28 uptake is significantly increased in the brainstem (with no [^18^F]FDG uptake increase) indicates the selectivity of this radiotracer for detecting neuroinflammatory responses. In addition, the increased [^18^F]FDG uptake in the cerebellum (with no [11C]PBR28 uptake increase) suggests an increased metabolic activity most likely driven by neuronal signaling with no significant microglial contribution. The differential impact of systemic inflammation on the brain was indicated by the observed simultaneous and individual decreases in [^18^F]FDG and [11C]PBR28 uptake in the primary motor and somatosensory cortices. Intriguingly, in addition to suppressed neuronal activity, these findings indicate a process of suppressed microglial activity. This observation is driven at least in part by the use of normalized radiotracer uptake in the analysis and deserves future examination in terms of its neurobiological interpretation and implication.

Of note, there is recent evidence for the presence of TSPO in brain vascular endothelial cells, cerebellar Purkinje cells, and neural stem/progenitor cells, indicating a broader range of cell populations expressing TSPO in the normal mouse brain (Betlazar et al, 2018). However, the contribution of these cellular sources of TSPO to the brain [^11^C]PBR28 uptake in the absence and presence of inflammation is presently unknown.

There are a few possible mechanisms underlying the impact of systemic inflammation on the brain (Pavlov & Tracey, 2015; Silverman et al, 2015; Pavlov et al, 2018; Capuron & Miller, 2011). In endotoxemia, direct activation of neuroinflammation by LPS is unlikely because of poor LPS penetration across the blood–brain barrier (Qin et al, 2007). However, pro-inflammatory cytokines, including TNF, IL-1b, and IL-6, whose circulating levels were significantly elevated have a demonstrated role in immune cell activation in the brain (Qin et al, 2007; Nadeau & Rivest, 1999). Very recently, a key role for TNF and TNF-STAT signaling was shown in astrocytic dysfunction and blood brain barrier impairment (Kim et al, n.d.). Underlying events may involve cytokine crossing of the blood–brain barrier by a saturable carrier-mediated mechanism, binding receptors at the surface of the endothelium of brain capillaries with the release of soluble mediators, such as prostaglandins and nitric oxide or acting via circumventricular organs that lack blood–brain barrier function (Qin et al, 2007; Capuron & Miller, 2011; Nadeau & Rivest, 1999; Pavlov et al, 2003). The expression of TLR4 and cytokine receptors on microglia and astrocytes provide a triggering mechanism for neuroinflammatory responses.

There is some skepticism about the clinical relevance of evaluating microglial activation using single tracer PET imaging, such as [^11^C]PBR28. As recently summarized, in Alzheimer’s disease, multiple sclerosis and amyotrophic lateral sclerosis increased [^11^C]PBR28 uptake is most likely a result of increased number of microglia rather than increased activation (Nutma et al, n.d.). Therefore, a dual or multi-tracer studies involving [^18^F]FDG and [^11^C]PBR28, and/or other specific ligands, capable of capturing alterations in the energy metabolism and the functional state of microglial activation may provide more complete evaluation.

The time point of our study microPET imaging study, i.e., 6 h after LPS administration was chosen based on data from previous postmortem studies demonstrating microglial activation and neuroinflammation at early stages of endotoxemia. The single time point could be considered as a limitation of our study. Further investigations evaluating brain region specific patterns of dual tracer uptake increases or decreases will be necessary to provide a more detailed characterization of brain alterations at different stages of endotoxemia.

The brain regulation of physiological functions is key to homeostasis. The hippocampus has a major role in the regulation of memory. The hypothalamus is an important regulator of the brain autonomic outflow that regulates several peripheral functions and its integration with the neuroendocrine regulation of physiological processes. The thalamus is a key region associated with processing sensory information. Further, the brainstem - the most evolutionarily conserved region of the brain - provides integrated reflex regulation of numerous physiological processes, including inflammation. Recently, it was demonstrated that a brainstem nucleus – the dorsal motor nucleus of the vagus controls peripheral TNF release through the vagus nerve-mediated *inflammatory reflex* (Kressel et al, 2020). The cerebellum contributes to motor and nonmotor functions, adaptive plasticity, and cognition. The primary somatosensory (S1) and primary motor (M1) cortices are reciprocally connected, and their interaction contribute to coordinated motor output. Changes in energy metabolism and neuroinflammation in these brain areas may result in cognitive impairment and other brain functional altercations. These changes may also lead to compromised regulations of peripheral physiological processes, which are specifically attributed to these brain areas. Thus, while systemic inflammation has an impact on the brain, in turn, altered brain function may contribute to and exacerbate peripheral pathology.

## Conclusions

Our observations indicate that neuroinflammatory alterations in endotoxemic mice, previously demonstrated utilizing postmortem evaluation (Silverman et al, 2015; Hoogland et al, 2015) can be reliably detected using dual tracer non-invasive microPET imaging. It is important to note that our observations reflect changes in the brain at early stages of endotoxemia and different patterns of brain alterations can be expected at later stages. These findings are of interest for further PET-based non-invasive evaluation of neuroinflammation in preclinical models and clinical settings of sepsis, and other lethal and debilitating disorders characterized by peripheral immune and metabolic dysregulation.

## Supplementary Information

**Additional file 1: Supplementary Fig. 1.** LPS administration results in significant increases in serum cytokine levels. Mice were injected with saline (*n*=8) or LPS (2 mg/kg, i.p.) (*n*=6) and euthanized 6h later. Blood was obtained through cardiac puncture and cytokines analyzed in the serum (***P=0.0007). See Methods for details.

**Additional file 2: Supplementary Fig. 2. **Brain individual tracer uptake decreases. Statistically significant clusters (*P*<0.001) of individual [^18^F]FDG and [^11^C]PBR28 decreases in primary and somatosensory motor cortices were subjected to post-hoc analysis of decreases in the same groups (saline, *n*=6 and LPS, *n*=6) of mice. **P=0.002; **P=0.007 ([^18^F]FDG **-** M1). See Methods for details.

**Additional file 3: Supplementary methods. **Serum cytokines analysis. Cytokines, including TNF, IL-6, IL-10, and IL-1b, were determined in serum using a cytokine panel detection kit (Invitrogen) following the manufacture’s recommendations.

## Data Availability

All data generated are included in this article and its supplementary information files. The datasets analyzed during the current study are available from the corresponding author on reasonable request.

## Abbreviations

([^11^C]PBR28: [^11^C]-peripheral benzodiazepine receptor, [methyl-^11^C]N-acetyl-N-(2-methoxybenzyl)-2-phenoxy-5-pyridinamine
CNS: Central nervous system
[^18^F]FDG: [^18^F] Fluoro-2-deoxy-2-D-glucose
IAW: Inveon Acquisition workflow
LPS: Lipopolysaccharide
microPET: Micro positron emission tomography
PMOD: Pixel-Wise Modeling
3D-OSEM: Three-dimensional ordered subsets-expectation maximization
TSPO: Translocator protein.
