# Supplementary figures and images for: A dual tracer [11C]PBR28 and [18F]FDG microPET evaluation of neuroinflammation and brain energy metabolism in murine endotoxemia
Source: Bioelectron Med. 2022 Nov 30;8:18. doi: 10.1186/s42234-022-00101-2 (PMC9710165; doi:10.1186/s42234-022-00101-2)

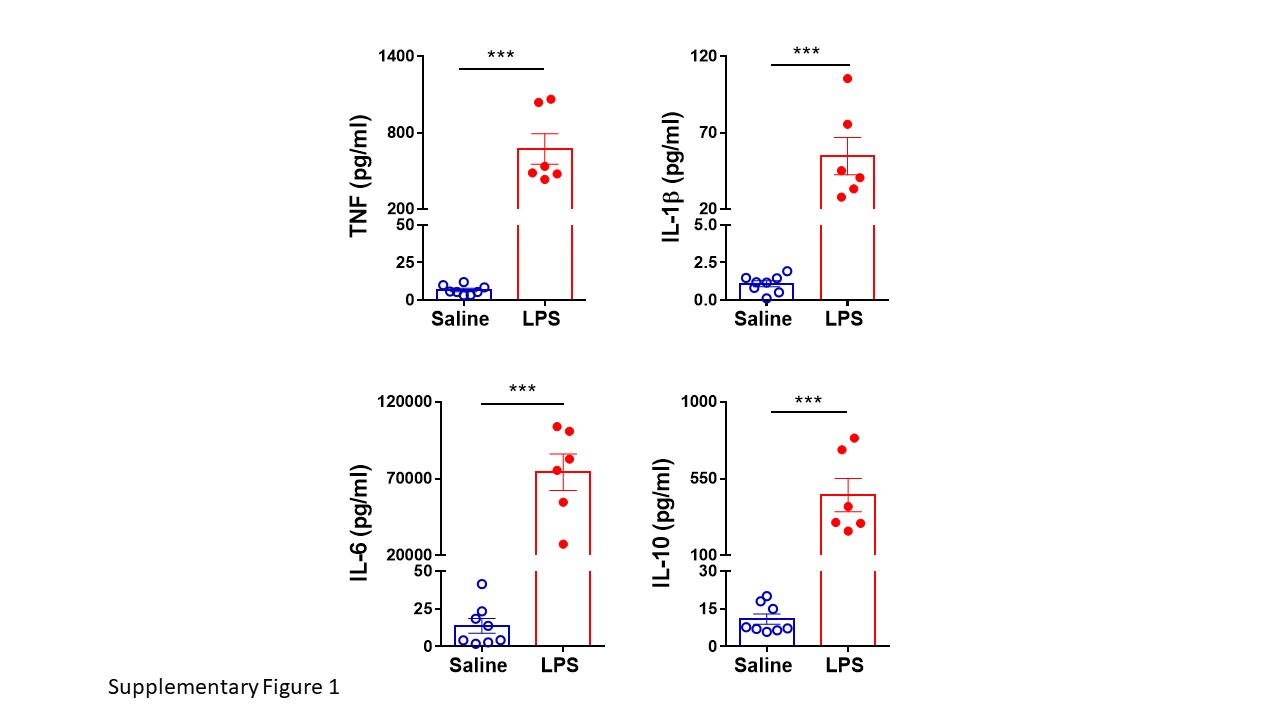

Supplement: Supplementary file 1 — Additional file 1: Supplementary Fig. 1. LPS administration results in significant increases in serum cytokine levels. Mice were injected with saline (n=8) or LPS (2 mg/kg, i.p.) (n=6) and euthanized 6h later. Blood was obtained through cardiac puncture and cytokines analyzed in the serum (***P=0.0007). See Methods for details. [file 42234_2022_101_MOESM1_ESM.jpg]

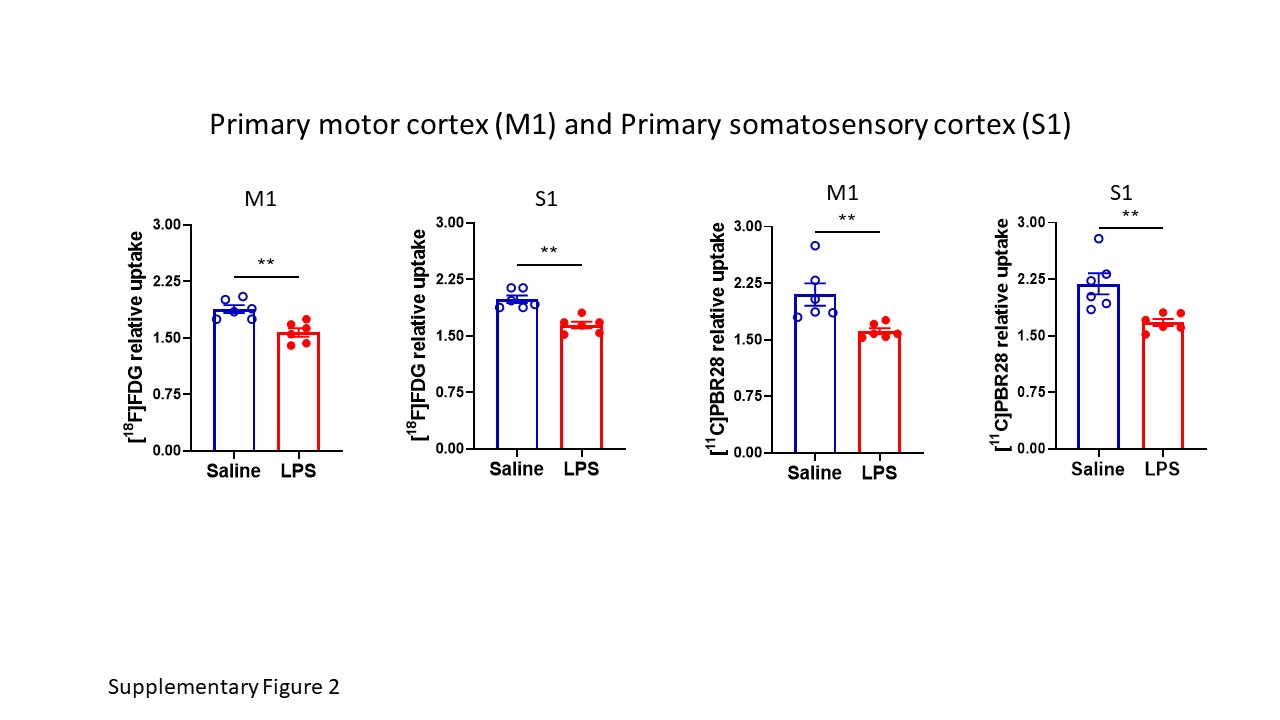

Supplement: Supplementary file 2 — Additional file 2: Supplementary Fig. 2. Brain individual tracer uptake decreases. Statistically significant clusters (P<0.001) of individual [18F]FDG and [11C]PBR28 decreases in primary and somatosensory motor cortices were subjected to post-hoc analysis of decreases in the same groups (saline, n=6 and LPS, n=6) of mice. **P=0.002; **P=0.007 ([18F]FDG - M1). See Methods for details. [file 42234_2022_101_MOESM2_ESM.jpg]
