# Supplementary material for: A dual tracer [11C]PBR28 and [18F]FDG microPET evaluation of neuroinflammation and brain energy metabolism in murine endotoxemia
Source: Bioelectron Med. 2022 Nov 30;8:18. doi: 10.1186/s42234-022-00101-2 (PMC9710165; doi:10.1186/s42234-022-00101-2)
Supplement: Supplementary file 3 — Additional file 3: Supplementary methods. Serum cytokines analysis. Cytokines, including TNF, IL-6, IL-10, and IL-1b, were determined in serum using a cytokine panel detection kit (Invitrogen) following the manufacture’s recommendations. [file 42234_2022_101_MOESM3_ESM.docx]

**Supplementary methods**

***Serum cytokines analysis***

Cytokines, including TNF, IL-6, IL-10, and IL-1β, were determined in serum using a cytokine panel detection kit (Invitrogen) following the manufacture’s recommendations.
